# Use of an Acellular Dermal Matrix Graft to Address a Contour Deformity in the Temporal Fossa: A Novel Application

**DOI:** 10.7759/cureus.5933

**Published:** 2019-10-17

**Authors:** Raghav Gupta, Nicholas C Oleck, Nitesh V Patel, Ira Goldstein

**Affiliations:** 1 Neurosurgery, Rutgers New Jersey Medical School, Newark, USA; 2 Plastic Surgery, Rutgers New Jersey Medical School, Newark, USA; 3 Neurosurgery, Lenox Hill Hospital, New York, USA

**Keywords:** cranioplasty, alloderm, implant, temporalis fossa, muscle atrophy, cosmetic revision

## Abstract

Cranioplasty of the frontotemporoparietal region is particularly challenging given the thin skin and musculature in this area, predisposing one to an increased risk of contour deformity and cosmetic dissatisfaction following surgery. Herein, we describe a 36-year-old male who initially presented with a gunshot wound (GSW) to the head and a right parietal skull fracture and underwent a revision of his cranioplasty procedure due to significant temporalis muscle atrophy, resulting in a sunken appearance of the right temporalis fossa following a craniectomy and multiple surgeries for hematoma evacuation. The patient underwent cranioplasty for definitive repair of his defect, and at follow-up, significant temporalis muscle atrophy resulted in a sunken appearance of the right temporalis fossa. A calcium phosphate bone substitute was used to fill the deformity, but dissolution and migration of the cement at follow-up necessitated a repeat cranioplasty procedure. Alloderm™ (Allergan Corp., Dublin, Ireland), an acellular dermal matrix derived from cadaveric skin, which has been previously used for dural repair, was successfully used in this study as a buffer between the skin and a cranioplasty implant to enhance cosmetic outcomes in a revision cranioplasty procedure following temporalis muscle atrophy.

## Introduction

Prosthetic materials utilized in cranioplasty commonly fall into one of three categories: metallic, ceramic, or polymer-based [1]. The advent of three-dimensional (3D) printing has led to the development of patient-specific prosthetic devices. These devices help achieve a precise fit for cranial defects, decrease operative time, and improve cosmetic outcomes [2]. In recent years, polyetheretherketone (PEEK) implants have become popular. These PEEK implants offer durability, radiolucency, and a precise fit, leading to improved structural and cosmetic outcomes [2].

While computer-aided implant designs provide a precise fit for cranial deficits, they do not address the issue of soft tissue coverage or muscular atrophy, the frequent factors associated with traumatic head injury repair [3]. Obtaining desirable cosmetic outcomes following cranioplasty of the frontotemporoparietal region of the cranium is particularly challenging. This is in part due to the thin nature of the skin and underlying musculature in this area, as well as the prominence and visibility of this region [4]. Failure of adequate reconstruction may lead to asymmetry, contour deformities, or a sunken appearance. In this report, we describe a novel technique in which an acellular dermal matrix graft, Alloderm™ (Allergan Corp., Dublin, Ireland), was layered over a frontotemporoparietal PEEK implant to address a contour deformity.

## Technical report

Initial presentation and cranioplasty

**Figure 1:**
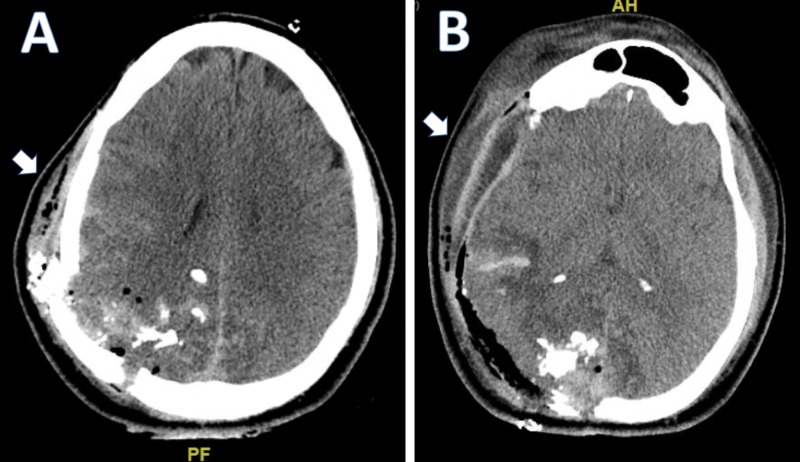
Initial frontotemporoparietal craniectomy pre-op (A) and post-op (B) CT imaging CT, computed tomography

The patient is a 36-year-old male who presented to the emergency department (ED) after suffering a gunshot wound (GSW) to the head. Upon arrival to the ED, computed tomography (CT) imaging revealed multi-compartmental intracranial hematomas and a parietal skull fracture. A right frontotemporoparietal craniectomy was performed for the evacuation of the intraparenchymal hematoma and removal of intraparenchymal bone fragments (Figure 1). 

**Figure 2:**
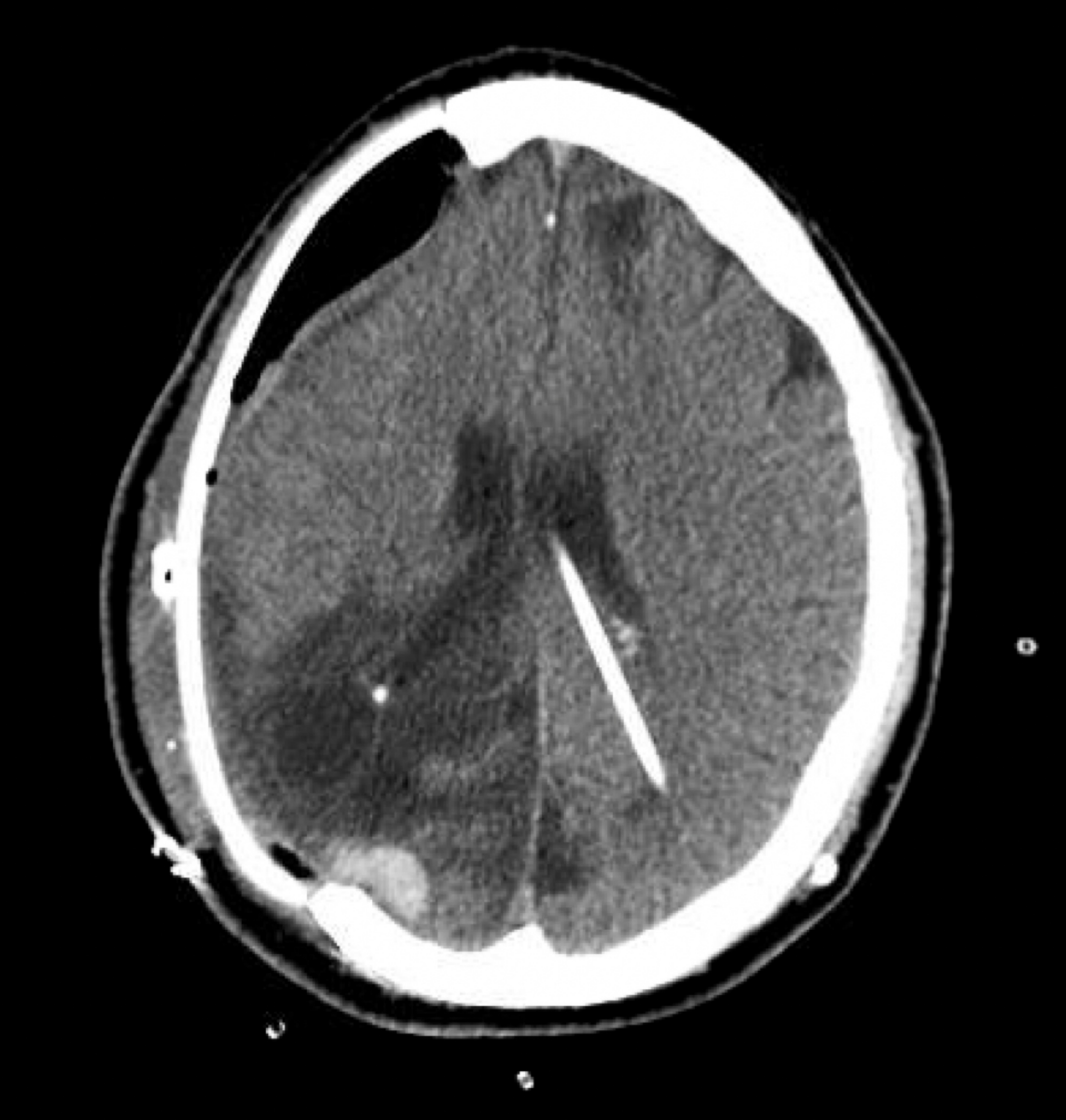
Primary cranioplasty surgery post-op CT imaging CT, computed tomography

Following a 23-day hospital stay, the patient’s neurological status improved, and he was discharged to a rehabilitation facility. Several weeks later, the patient once again had an episode of neurological decline and workup showed the formation of another intraparenchymal hematoma (likely secondary to a venous infarct) requiring surgical evacuation. A large subgaleal fluid collection was also noted. Several months after this hospital stay, the patient underwent cranioplasty for definitive repair of his cranial defect using a custom-fitted 3D PEEK implant (Stryker Corp., Kalamazoo, Michigan; Figure 2).

A left-side ventriculoperitoneal shunt was also placed and the patient was noted to have hydrocephalus. The procedure was well tolerated by the patient and he was discharged after a short hospital stay.

Initial cranioplasty revision

Although the cranioplasty with PEEK implant achieved excellent cranial contour, at follow-up, the patient was noted to have a significant “hollowing out” and sunken appearance of the right temporalis fossa due to significant temporalis muscle atrophy. Cranioplasty augmentation was performed to address this deficit. During this initial reconstruction, the temporalis muscle was mobilized, and a calcium phosphate bone substitute was used to fill the space just rostral to the zygoma and posterior to the right orbit. A flat contour was attained, and the temporalis was laid over the surface of the bone substitute to minimize any step-off between the cement and the surrounding skull.

Cranioplasty revision with layered Alloderm™

**Figure 3:**
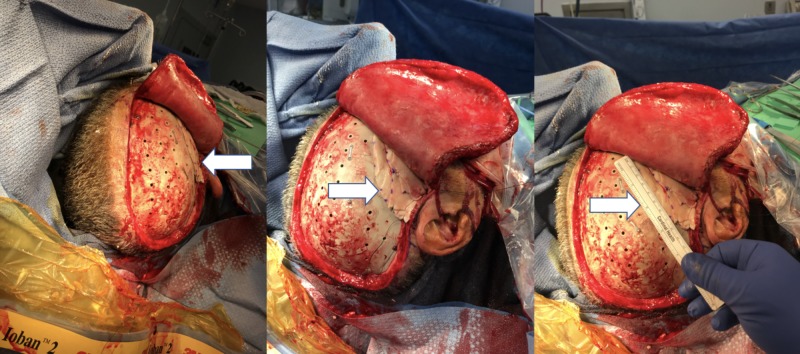
Subsequent cranioplasty intra-operative images: use of an acellular cadaveric dermal matrix, Alloderm™, to correct a contour deformity in the temporal fossa following a frontotemporoparietal cranioplasty

At the next follow-up, two months later, the patient was noted to have persistent asymmetry and fullness at the site of the cement application. A CT scan demonstrated a small degree of dissolution and migration of the cement, resulting in a persistent protrusion at that point as well as discomfort. The decision was made to proceed with a second cranioplasty revision. The previous right frontotemporoparietal craniectomy incision was reopened. The cement was subsequently removed; after considering several soft tissue grafting options, Alloderm™ was chosen to fix the defect. A thick sheet of Alloderm™ was cut into multiple sheets that were then sutured together to approximate the contour of the temporalis muscle (Figure 3).

**Figure 4:**
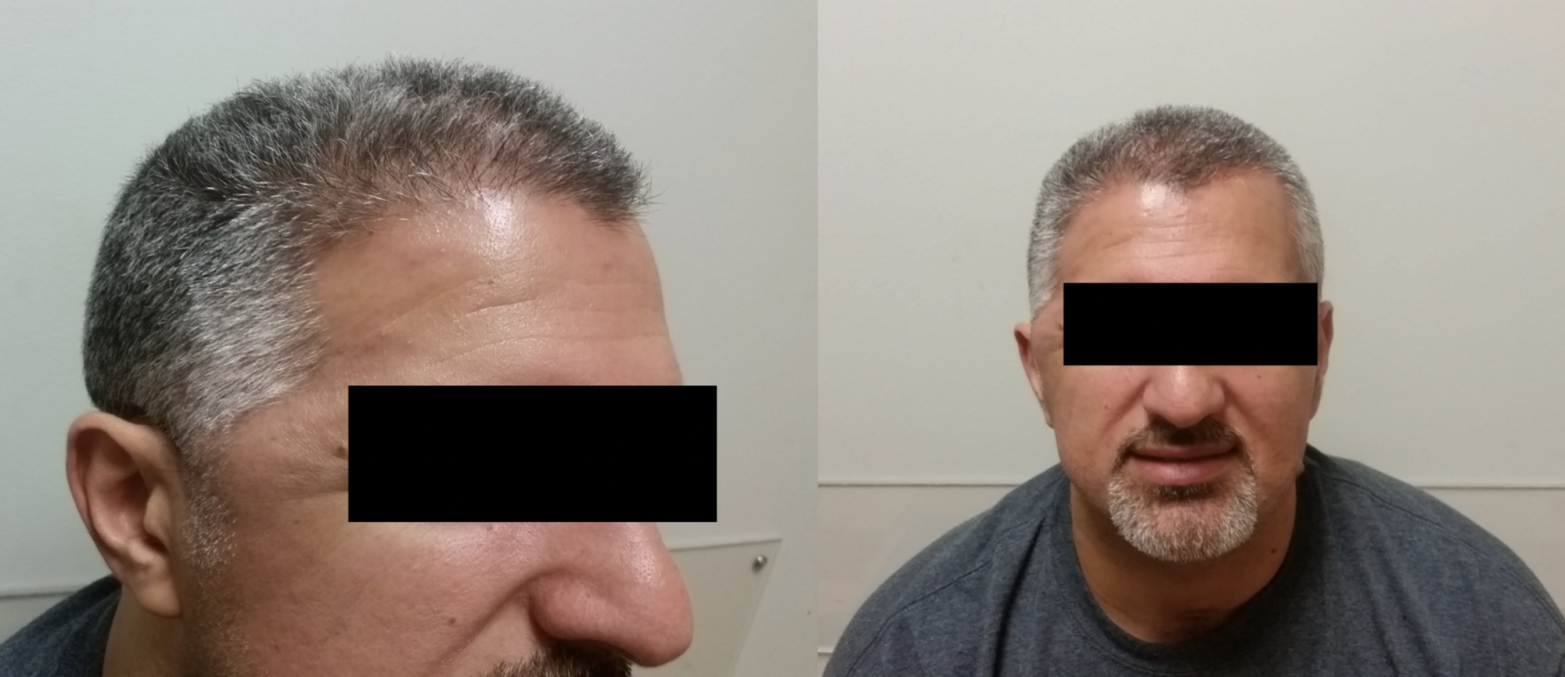
Office follow-up cosmetic outcome At clinical follow-up, the previous temporal fossa contour deformity was significantly improved.

To accomplish this, these sheets were layered with the thickest point just posterior to the orbit and just superior to the zygoma, and the thinnest point at the posterior and rostral margin. The margins were then sutured to soft tissue just posterior to the orbital ridge at the level of the galea, and soft tissue just rostral to the ear at the level of the zygoma. The scalp was reflected back, and the appearance of symmetry was demonstrated. At clinical follow-up, palpation posterior to the orbital ridge demonstrated that the hollowing out that had been previously present, was now filled out well (Figure 4).

A small bump above the zygoma was present, indicating slight migration of the layered Alloderm™ inferiorly.

## Discussion

The use of Alloderm™, an acellular dermal matrix derived from human cadaveric skin, has been well-documented in the neurosurgical literature. It has been used specifically for dural repair when primary closure or autologous site harvest is not possible. Gaynor et al. have previously presented data on its role in sellar floor repair following transsphenoidal surgery for pituitary adenomas in a series on 429 patients [5]. Agag et al. have also reported on its role in the repair of meningomyelocele, encephalocele, and cerebrospinal fluid (CSF) fistulas [6]. Its use in cranioplasty, however, has not been clearly elucidated. In a recent case report, Singh et al. described the use of Alloderm™ in covering a titanium implant in the frontal bone position. The authors argued that given the thinness of the frontalis muscle and soft tissue coverage in their index patient, the Alloderm™ served to prevent mesh palpability and cosmetic dissatisfaction [7].

In the present study, we describe a novel technique in which Alloderm™ was layered on top of an existing custom-fitted 3D PEEK implant used in a frontotemporoparietal cranioplasty to address an underlying “sunken” deformity in the temporal fossa. In this second cranioplasty revision procedure, the Alloderm™ allowed for approximation of the contour of the temporalis muscle, which was severely atrophied. The Alloderm™ was used to replace a calcium phosphate bone substitute that had dislodged, leading to a protrusion in the region posterior to the superior orbital ridge and superior to the zygomatic arch. Postoperatively, the deformity was no longer present, and the scalp was symmetric, bilaterally. The use of Alloderm™ implants in the frontotemporal region during cranioplasty may provide a buffer given that this area is particularly susceptible to thinner skin and muscle atrophy, as was present in our patient [8]. Additionally, given that defects in this region can lead to significant disfigurement, Alloderm™ may be used to enhance postoperative cosmetic outcomes. The advantages of using Alloderm™ have been clearly delineated in the past. The graft is composed of an acellular scaffold that predominantly consists of collagen and elastic fibers akin to an extracellular matrix (ECM) [9]. These may potentially enable native tissue ingrowth and revascularization of the Alloderm™ graft [9]. In addition, Alloderm™ is immunologically inert which prevents rejection and/or foreign body reactions [7]. However, the advantages of using Alloderm™ in cranioplasty procedures should be carefully weighed against its cost, particularly given that Gaynor et al. have previously cited the cost of 8 cm^2^ of Alloderm™ to be approximately $400 USD, as well as the risk for disease transmission [5]. Nonetheless, the ease with which Alloderm™ can be utilized for this purpose should certainly be factored in, given the complexity associated with other more elaborate alternatives such as custom-fitted silicone implants and muscle transplantation.

## Conclusions

In this report, we describe a novel technique in which Alloderm™, an acellular cadaveric dermal matrix, was used to correct a contour deformity in the temporal fossa following a frontotemporoparietal cranioplasty performed with a PEEK implant. We believe this substance may be a simple solution in patients with thin or atrophied temporalis muscle and can serve as a buffer between the skin and the cranioplasty implant to enhance cosmetic outcomes.
